# Translation Elongation Factor Tuf of *Acinetobacter baumannii* Is a Plasminogen-Binding Protein

**DOI:** 10.1371/journal.pone.0134418

**Published:** 2015-07-31

**Authors:** Arno Koenigs, Peter F. Zipfel, Peter Kraiczy

**Affiliations:** 1 Institute of Medical Microbiology and Infection Control, University Hospital of Frankfurt, Frankfurt, Germany; 2 Department of Infection Biology, Leibniz Institute for Natural Product Research and Infection Biology, Jena, Germany; 3 Friedrich Schiller University, Jena, Germany; University of North Dakota School of Medicine and Health Sciences, UNITED STATES

## Abstract

*Acinetobacter baumannii* is an important nosocomial pathogen, causing a variety of opportunistic infections of the skin, soft tissues and wounds, urinary tract infections, secondary meningitis, pneumonia and bacteremia. Over 63% of *A*. *baumannii* infections occurring in the United States are caused by multidrug resistant isolates, and pan-resistant isolates have begun to emerge that are resistant to all clinically relevant antibiotics. The complement system represents the first line of defense against invading pathogens. However, many *A*. *baumannii* isolates, especially those causing severe bacteremia are resistant to complement-mediated killing, though the underlying mechanisms remain poorly understood. Here we show for the first time that *A*. *baumannii* binds host-derived plasminogen and we identify the translation elongation factor Tuf as a moonlighting plasminogen-binding protein that is exposed on the outer surface of *A*. *baumannii*. Binding of plasminogen to Tuf is at least partly dependent on lysine residues and ionic interactions. Plasminogen, once bound to Tuf can be converted to active plasmin and proteolytically degrade fibrinogen as well as the key complement component C3b. Thus, Tuf acts as a multifunctional protein that may contribute to virulence of *A*. *baumannii* by aiding in dissemination and evasion of the complement system.

## Introduction

*Acinetobacter* (*A*.) *baumannii* is emerging as an important opportunistic pathogen and responsible for 2–10% of Gram-negative nosocomial infections [1]. The species *A*. *baumannii* has only been designated in 1986 and while other *Acinetobacter* species are frequently isolated from soil or water [2], the natural habitat of *A*. *baumannii* remains unknown. To date, *A*. *baumannii* is found almost exclusively in healthcare settings, particularly in intensive care units [3]. Clinical manifestations of *A*. *baumannii* infections comprise skin and soft tissue infections, wound infections, urinary tract infections and secondary meningitis. Infections associated with the highest mortality rates include ventilator-associated pneumonia and bacteremia [4]. *A*. *baumannii* is exceptionally tolerant toward desiccation stress [5] and resistant to the most commonly prescribed antibiotics [6], allowing the pathogens to persist in the hospital environment. As of 2013, 63% of *Acinetobacter* infections occurring in the United States were caused by multidrug resistant isolates according to the CDC [7]. Of particular concern is the emergence of pan-resistant *A*. *baumannii* strains, which are resistant to all clinically relevant antibiotics and pose an enormous challenge to clinicians [8]. While multidrug resistance remains a prevalent topic when discussing *A*. *baumannii* infections, a number of virulence factors contribute to its pathogenic potential, however many of them are not well understood.

The complement system is a central component of the innate immune system and plays numerous roles in defense and homeostasis [9]. Complement is activated through three canonical pathways. Antibody-antigen complexes activate the classical pathway, while recognition of specific carbohydrates (e.g. mannan) results in activation of the lectin pathway. By contrast, activation of the alternative pathway occurs spontaneously. Activation of either pathway results in the formation of complexes known as C3 convertases and subsequent proteolytic cleavage of the central complement component C3. The larger cleavage fragment, C3b is deposited on the surface of invading pathogens, leading to opsonization [10] and marking pathogens for phagocytosis, while the smaller cleavage fragment, C3a displays antimicrobial activity and serves as a powerful chemoattractant for phagocytes [11]. When C3b binds to surface attached C3 convertases, it alters the substrate specificity of the convertase from C3 to C5. These C5 convertases cleave C5, thereby initiating the terminal pathway of complement activation, resulting in formation of the terminal complement complex (TCC) [12]. The TCC forms a lytic pore and destabilizes the bacterial membrane, leading to direct killing of invading pathogens [13].

Plasminogen is a 92-kDa glycoprotein, synthesized in the liver and present in human serum in a concentration of approximately 2.4 μM. Additionally, plasminogen is also found in many extravascular fluids. The inactive proenzyme consists of an N-terminal preactivation peptide, five lysine-binding, disulfide-bonded kringle domains and a serine protease domain [14]. Proteolytic cleavage of plasminogen by activators, such as the endogenous tissue-type plasminogen activator and urokinase-type plasminogen activator, results in the generation of plasmin, the active serine protease [15]. Plasmin is an important component of the human fibrinolytic system and exhibits a relatively low substrate specificity. In addition to the physiological substrate fibrinogen, plasmin degrades components of the extracellular matrix such as fibronectin, vitronectin, laminin, heparan sulfate proteoglycans and inactive precursors of various matrix metalloproteases. Furthermore, plasmin is able to cleave the complement components C3b and C5 and the proteolytically inactive zymogen plasminogen enhances complement factor I-mediated inactivation of C3b in the presence of factor H [16]. Plasmin(ogen) thus functions as a complement regulator. An ever increasing number of diverse human pathogens recruit plasminogen to their surface, including Gram-positive bacteria such as *Streptococcus pneumonia* [17] and *Staphylococcus aureus* [18], Gram-negative bacteria like *Pseudomonas aeruginosa* [19], *Haemophilus influenzae* [20] and *Helicobacter pylori* [21], spirochetes such as *Leptospira interrogans* [22, 23] and *Borrelia burgdorferi* [24] as well as the invasive yeast *Candida albicans* [25]. These examples underline, that binding of plasminogen is a strategy employed by various pathogenic microorganisms to disseminate and persist in the human host.

The translation elongation factor Tuf is a ubiquitous, highly conserved protein that is usually located in the cytoplasm. Cytoplasmic Tuf binds to aminoacyl-tRNAs and transports the latter to the ribosome where it controls the elongation of polypeptide chains. Tuf also seems to function as a chaperone, supporting folding and renaturation of other proteins [26]. Moreover, it has been demonstrated that Tuf of *Bacillus subtilis* interacts with the actin-like MreB protein, playing a role in bacterial cell shape maintenance [27]. In several pathogenic microorganisms and in addition to its intracellular function, Tuf is located on the bacterial surface, where it functions as a moonlighting protein and interacts with various host proteins. The Tuf proteins of *Pseudomonas aeruginosa*, *Streptococcus pneumoniae* and *Leptospira interrogans* bind both plasminogen and factor H, the key complement regulator of the alternative pathway [28–30]. Tuf of *Mycobacterium tuberculosis* binds plasminogen and fibronectin [31, 32]. It has recently been demonstrated, that Tuf of *A*. *baumannii* is able to interact with fibronectin as well [33]. Here, we show for the first time that *A*. *baumannii* interacts with human plasminogen and identify Tuf as a plasminogen-binding protein.

## Materials and Methods

### Bacterial strains and culture conditions

*A*. *baumannii* type strain ATCC 19606 was grown at 37°C in lysogeny broth [34]. Bacterial cells were counted using a Kova counting chamber (Hycor Biomedical, Indianapolis, IN, USA). *Legionella pneumophila* (clinical isolate from tracheal secretion, serotype 1) was grown at 37°C on charcoal yeast extract agar (Oxoid, Wesel, Germany). *Escherichia coli* JM109 cells (Promega) used for heterologous expression of Tuf were grown in yeast tryptone broth at 37°C.

### Proteins and antisera

Human glu-plasminogen was obtained from Haematologic Technologies (Essex Junction, VT, USA). Plasminogen was activated to plasmin using urokinase plasminogen activator (uPA) from Merck Millipore, Darmstadt, Germany. Both the chromogenic substrate S-2251 (D-Val-Leu-Lys *p*-nitroanilide dihydrochloride) and fibrinogen were purchased from Sigma-Aldrich (Steinheim, Germany). Purified C3b was obtained from Complement Technology, Tyler, TX, USA. *A*. *baumannii* Tuf was detected using a polyclonal rabbit antiserum raised against *Streptococcus pneumoniae* Tuf [29]. C3 and fibrinogen polyclonal antisera were purchased from Acris Antibodies (Herford, Germany). The monoclonal hexahistidine antibody was obtained from GE Healthcare (Munich, Germany). Horseradish peroxidase (HRP)-conjugated immunoglobulins were purchased from Dako (Hamburg, Germany) and Alexa Fluor 488-conjugated anti-rabbit immunoglobulins from Life Technologies (Darmstadt, Germany).

### Generation of recombinant, polyhistidine-tagged proteins

The Tuf encoding gene of *A*. *baumannii* type strain ATCC 19606 (ORF HMPREF0010_03765) was amplified by PCR from genomic DNA using primers FP Abau TufB-BamHI (5’-cgtaaacgaggaagggatccatggctaaagccaagtttgaacg-3’) and RP Abau TufB-HindIII (5’-gagacgtaattcgtcactatattaagcttatgcagttactttagc-3’). The gene encoding Tuf of *L*. *pneumophila* was amplified by PCR from genomic DNA using primers FP Leg TufB (5’-gttaacgaggttggatccatggcgaaggaaaaatttgaacgtaag-3’) and RP Leg TufB (5’-taatattttgattgctactcaagctttttatgcagttactttagc-3’). The PCR products were cloned into the pQE-30 Xa expression vector (Qiagen, Hilden, Germany). The resultant plasmids, pQE-Tuf_Ab_ and pQE-Tuf_Lp_ were sequenced to ensure no mutations had been introduced during PCR or the subsequent cloning process. Recombinant Tuf proteins were produced in *E*. *coli* strain JM109 (Promega, Mannheim, Germany) upon induction with isopropyl-β-D-thiogalactopyranoside (IPTG). Cells were harvested, and lysed with a MICCRA D-9 dispersion device (Art Prozess- & Labortechnik, Mullheim, Germany) in lysis buffer containing 10 mM Imidazole, 300 mM NaCl, 50 mM NaH_2_PO_4_ and 1 mg/ml lysozyme (pH 8.0). Following centrifugation to clear cell debris, proteins were purified using Amintra Ni-NTA resin (Expedeon, Cambridge, UK). 10% Tris/Tricine SDS-PAGE followed by silver staining was used to assess purity of the samples. Protein concentrations were determined by bicinchoninic acid protein assay (Life Technologies, Darmstadt, Germany). Recombinant BBA70, used as a positive control for fibrinogen and C3b degradation assays was produced as previously described [35].

### Far Western blotting

Recombinant proteins (500 ng each) were separated by reducing 10% Tris/Tricine SDS-PAGE and transferred to nitrocellulose membranes. Following blocking with 5% nonfat dry milk powder in TBS containing 0.1% Tween 20 (TBS-T), membranes were overlaid with 20 μg/ml plasminogen in PBS at room temperature for 1 h. After three wash steps with 0.2% TBS-T, plasminogen bound to denatured *Acinetobacter* proteins was detected with a polyclonal antiserum (diluted 1:1,000) raised against human plasminogen, followed by horseradish peroxidase (HRP)-conjugated anti-goat immunoglobulins (diluted 1:1,000) (Dako, Hamburg, Germany). Immune complexes were visualized with tetramethylbenzidine (TMB).

### SDS-PAGE, Western blotting and silver staining

500 ng of recombinant proteins or BSA (negative control) were separated by reducing 10% Tris/Tricine SDS-PAGE and transferred to nitrocellulose membranes. Following protein transfer, membranes were blocked with 5% nonfat dry milk powder in TBS containing 0.1% Tween 20. After three wash steps with 0.1% TBS-T, membranes were probed with a monoclonal hexahistidine antibody (diluted 1:3,000) followed by horseradish peroxidase (HRP)-conjugated anti-mouse immunoglobulins (diluted 1:1,000). Immune complexes were visualized with tetramethylbenzidine (TMB). Alternatively, 500 ng of recombinant proteins or BSA were subjected to 10% Tris/Tricine SDS-PAGE and gels were silver stained.

### Enzyme-linked immunosorbent assay (ELISA)

MaxiSorp 96-well microtiter plates (Nunc) were coated with 100 μl of recombinant proteins or BSA (5 μg/ml) in PBS at 4°C overnight with gentle agitation. Following three wash steps with PBS containing 0.05% (v/v) Tween 20 (PBS-T), wells were blocked with blocking buffer III BSA (AppliChem, Darmstadt, Germany) for 2 h at RT. Wells were washed three times with PBS-T and incubated with 100 μl plasminogen (10 μg/ml) at RT for 1 h. Following incubation, wells were washed thoroughly with PBS-T incubated with a polyclonal goat antiserum raised against human plasminogen (1:1,000) for 1 h at RT. After washing three times with PBS-T, wells were incubated with HRP-conjugated anti-goat immunoglobulins (1:2,000) at RT for 1 h. The reaction was developed with *o*-phenylenediamine (Sigma-Aldrich, Steinheim, Germany) and the absorbance was measured at 490 nm using an ELISA reader (PowerWave HT, Bio-Tek Instruments, Winooski, VT, USA, with Gen5 software from Bio-Tek Instruments, Winooski, VT, USA).

The role of lysine residues in plasminogen binding was investigated by addition of increasing amounts of the lysine analog tranexamic acid (Sigma-Aldrich). The effect of increasing ionic strength on the Tuf-plasminogen interaction was determined by incubation with increasing concentrations of NaBr. To determine dose-dependency of plasminogen binding and calculate the dissociation constant, immobilized Tuf was incubated with increasing amounts of plasminogen.

### Plasminogen binding assay

Late log-phase *A*. *baumannii* cells (2 x 10^9^) were harvested by centrifugation at 5000 x g and washed twice in PBS. Following sedimentation, cells were resuspended in PBS containing 20 μg/ml plasminogen and incubated for 1 h at RT. Cells were then washed four times with PBSAT (PBS containing 0.05% (v/v) Tween20 and 0.02% (w/v) sodium azide) to remove all unbound protein. Proteins bound to the surface of *A*. *baumannii* were then eluted for 15 min using 0.1 M Glycine pH 2.0. The last wash fraction and the eluate fraction were retained and separated by 10% Tris/Tricine SDS-PAGE. Following transfer of proteins to a nitrocellulose membrane, plasminogen was detected with a polyclonal plasminogen antiserum.

### Plasminogen activation assay

Activation of Tuf bound plasminogen to plasmin was investigated using the chromogenic substrate D-Val-Leu-Lys-*p*-nitroanilide dihydrochloride (S-2251, Sigma-Aldrich). MaxiSorp 96-well microtiter plates (Nunc) were coated with 100 μl of recombinant proteins or BSA (5 μg/ml) in PBS at 4°C overnight. Wells were blocked with blocking buffer III BSA (AppliChem) for 2 h at RT and after washing with PBS-T, plasminogen (10 μg/ml) was added. Following incubation for 1 h at RT, wells were washed three times with PBS-T and incubated with 96 μl of a reaction mixture containing 50 mM Tris/HCl, pH 7.5, 300 mM NaCl, 0.003% Triton X-100, and 0.3 mg/ml S-2251. Finally, 4 μl of 2.5 μg/ml urokinase plasminogen activator (uPA) were added to activate bound plasminogen to plasmin. Microtiter plates were then incubated at 37°C and absorbance was measured every 30 mins at 405 nm for a period of 18 h. In controls, either plasminogen or uPA were omitted from the reaction mixtures, or plasminogen was added together with 50 mM tranexamic acid.

### Fibrinogen degradation assay

5 μg/ml of recombinant proteins or gelatin were immobilized in PBS on MaxiSorp 96-well microtiter plates (Nunc) over night at 4°C. After washing with PBS-T, wells were blocked with 0.1% (w/v) gelatin in PBS for 2 h at RT. Wells were washed with PBS-T and incubated with 10 μg/ml plasminogen at RT for 1 h. Following three wash steps with PBS-T, 93.5 μl of a reaction mixture was added, containing 50 mM Tris/HCl, pH 7.5 and 20 μg/ml fibrinogen. To activate bound plasminogen to plasmin, 6.5 μl uPA (2.5 μg/ml) was added. Microtiter plates were incubated at 37°C and aliquots were taken at different time intervals. Reactions were stopped by addition of SDS-PAGE sample buffer and separated by 10% Tris/Tricine SDS-PAGE. Following transfer to nitrocellulose membranes, fibrinogen and its degradation products were visualized using a polyclonal goat antiserum (1:1,000) raised against fibrinogen (Acris) and HRP-conjugated anti-goat immunoglobulins (Dako) (1:1,000).

### C3b degradation assay

Degradation of C3b by Tuf-bound plasminogen was assayed in a fashion similar to the fibrinogen degradation assay described above. Briefly, immobilized Tuf proteins or gelatin (10 μg/ml) were incubated with plasminogen (20 μg/ml) and after several wash steps, 93.5 μl of a reaction mixture consisting of 50 mM Tris/HCL, pH 7.5 and 20 μg/ml C3b was added to the wells. Plasminogen was activated to plasmin by addition of 6.5 μl uPA (2.5 μg/ml). Microtiter plates were incubated at 37°C and aliquots were taken at the indicated time intervals. Samples were separated by 10% Tris/Tricine SDS-PAGE and transferred to nitrocellulose membranes. Membranes were then probed with a polyclonal goat antiserum raised against human C3 (Acris) (diluted 1:1,000), followed by HRP-conjugated anti-goat Immunoglobulins (Dako) (diluted 1:1,000). Antigen-antibody complexes were visualized with TMB.

### Flow cytometry

To assess surface exposure of Tuf, viable *A*. *baumannii* cells (5 x 10^8^) were resuspended in FACS-buffer (1% (w/v) BSA in PBS) and incubated for 1 h at 4°C. Following incubation, a polyclonal antiserum raised against *S*. *pneumoniae* Tuf [29], which also detects Tuf proteins of other bacterial species, was used to detect *A*. *baumannii* Tuf (diluted 1:10). Cells were incubated with the antiserum for 1 h at RT. After several wash steps with PBS, cells were incubated with an anti-rabbit Alexa Fluor 488-conjugate (diluted 1:25) for 30 min at RT. Cells were washed three times with PBS and fixated with 3.75% (w/v) paraformaldehyde (PFA) in PBS. After two more wash steps, cells were resuspended in PBS and samples were assayed using a FACSCalibur flow cytometer (BD Biosciences, Heidelberg, Germany).

### Statistical analysis

One-way ANOVA followed by Bonferroni’s multiple comparisons test was performed using GraphPad Prism version 6.05 for Windows, GraphPad Software, La Jolla, CA, USA. Results were deemed statistically significant for *p* values ≤ 0.05.

## Results

### *A*. *baumannii* binds human plasminogen

**Fig 1:**
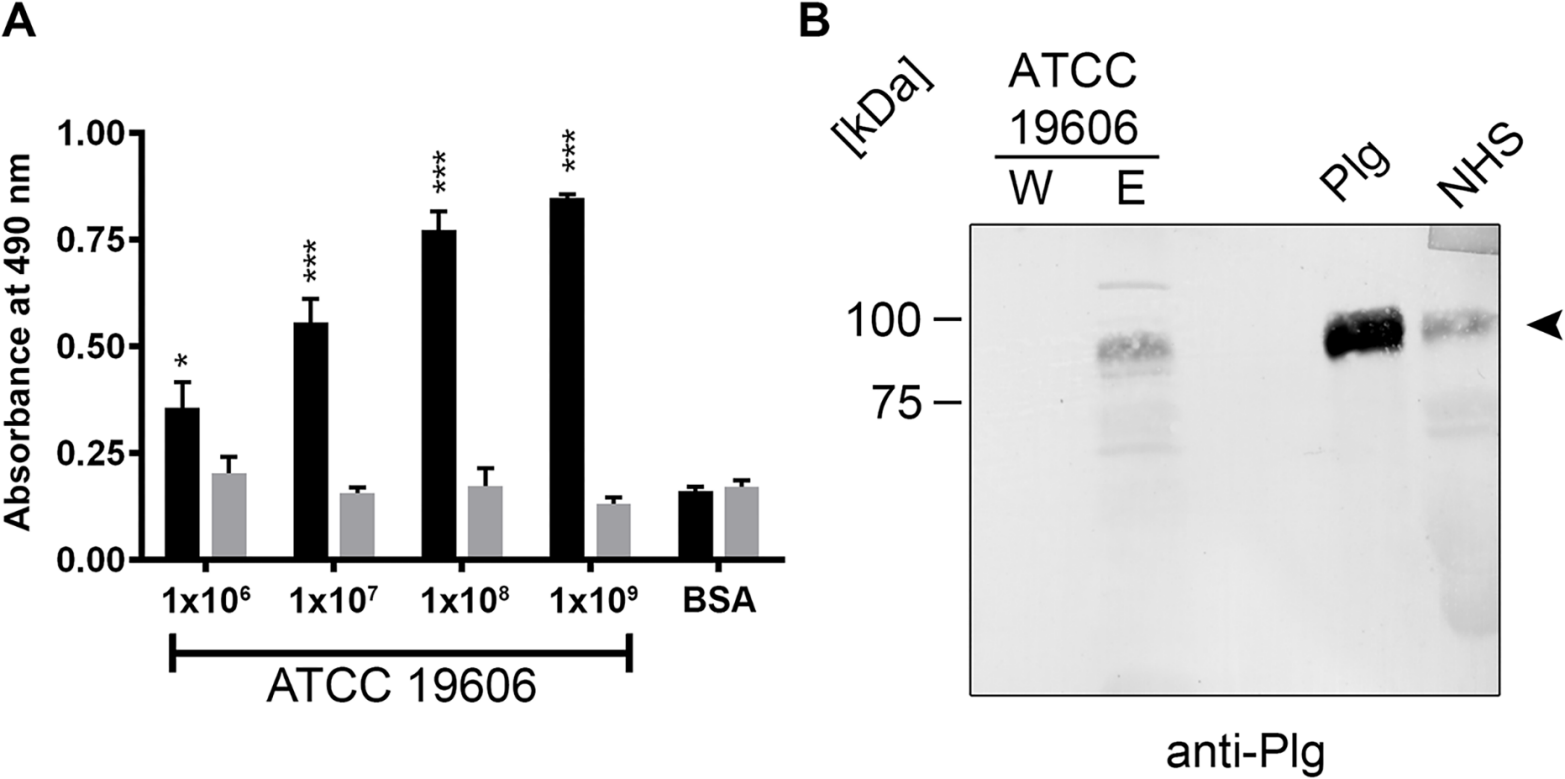
*A*. *baumannii* ATCC 19606 binds plasminogen. (A) Binding of plasminogen (10 μg/ml) to increasing numbers of *A*. *baumannii* cells was analyzed by whole cell ELISA. Bound plasminogen was detected using a polyclonal plasminogen antiserum. BSA was used as a control for nonspecific binding. Black bars represent plasminogen binding, gray bars represent background signals in the absence of plasminogen. Data represent mean values from at least three independent experiments, each performed in triplicate. Error bars represent standard deviation. *, p ≤ 0.05 and ***, p ≤ 0.001, one-way ANOVA with Bonferroni post hoc test. (B) Binding of plasminogen (20 μg/ml) to viable *A*. *baumannii* cells. 2 x 10^9^ cells were incubated with plasminogen. Following incubation, cells were washed thoroughly and bound proteins were eluted. The last wash fraction and eluate fraction were separated via SDS-PAGE. Proteins were transferred to a nitrocellulose membrane and probed with a polyclonal plasminogen antiserum. Purified plasminogen (500 ng) and NHS (2 μl of a 1:10 dilution) served as controls. Arrowhead indicates plasminogen with a molecular mass of 92 kDa.

To determine whether *A*. *baumannii* binds human plasminogen, increasing amounts of *A*. *baumannii* cells (type strain ATCC 19606) were immobilized onto microtiter plates and incubated with 10 μg/ml plasminogen. BSA served as a control for unspecific binding. After several wash steps, plasminogen bound to *A*. *baumannii* cells was detected with a polyclonal plasminogen antiserum. Significant binding was observed when using 1 x 10^6^ cells and signal strength increased, when increasing numbers of *A*. *baumannii* cells were immobilized (Fig 1A). Next, we sought to analyze binding of plasminogen to viable *A*. *baumannii* cells. 2 x 10^9^ bacterial cells were incubated with 20 μg/ml purified human plasminogen. After incubation, cells were washed thoroughly and bound plasminogen was eluted. The last wash fraction and the eluate fraction were retained and separated via SDS-PAGE. Following transfer to a nitrocellulose membrane, the membrane was probed with a polyclonal plasminogen antiserum. A signal was obtained for the eluate fraction (E) but not for the wash fraction (W), indicating that viable *A*. *baumannii* cells were able to bind human plasminogen (Fig 1B).

### Elongation factor Tuf of *A*. *baumannii* binds human plasminogen

**Fig 2:**
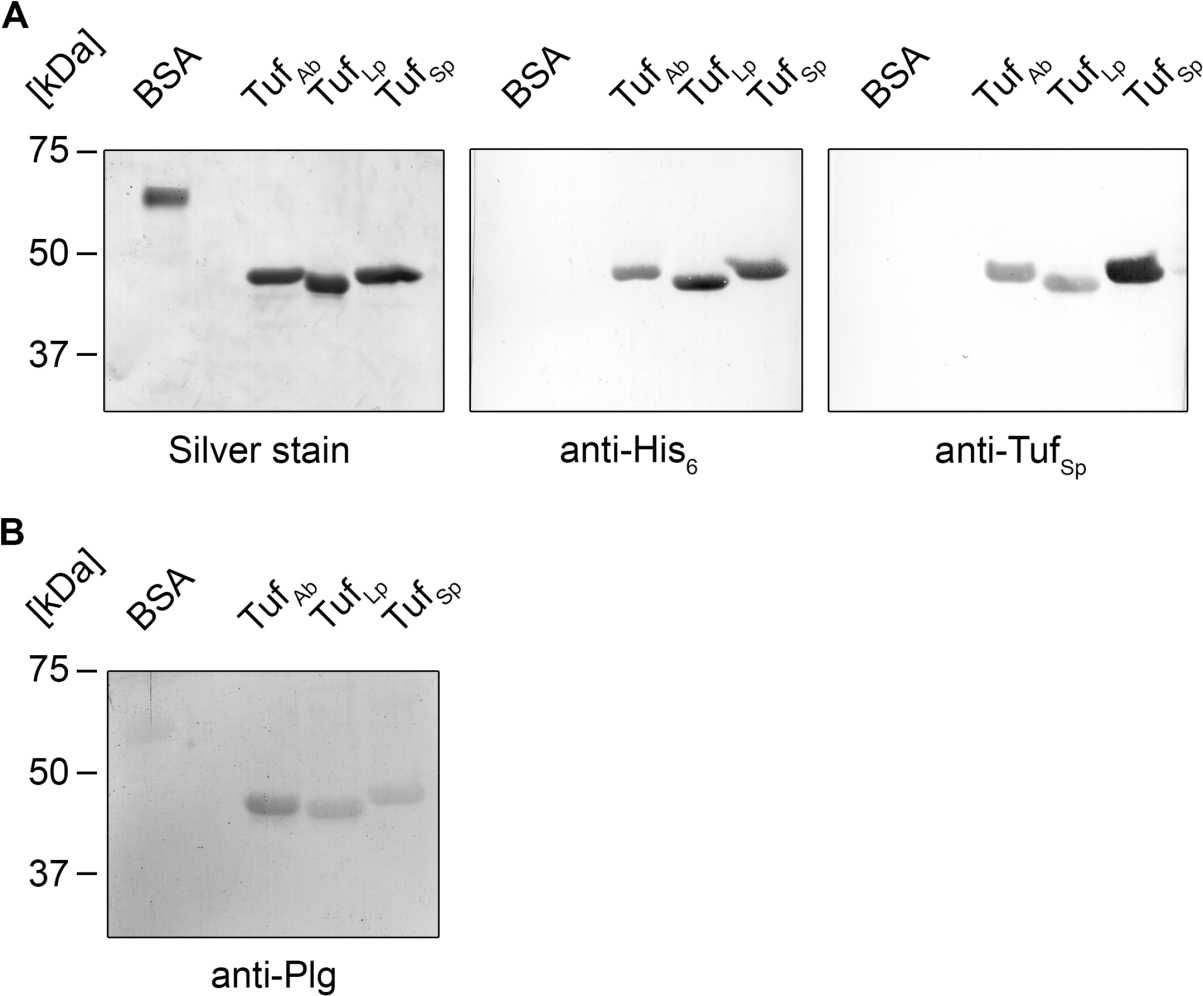
Recombinant Tuf of *A*. *baumannii* binds plasminogen. (A) Purity of the recombinant, hexahistidine-tagged proteins was assessed by silver staining (left panel) and Western blotting using a monospecific antibody raised against the hexahistidine-tag (anti-His_6_, middle panel). Western blot experiments using a polyclonal antiserum raised against Tuf of *S*. *pneumonia* (anti-Tuf_Sp_, right panel) revealed that this antiserum also reacts with Tuf proteins from *A*. *baumannii* (Tuf_Ab_) and *L*. *pneumophila* (Tuf_Lp_), making it suitable for detection of these proteins in subsequent experiments. (B) Binding of plasminogen (20 μg/ml) to purified Tuf proteins. Far Western blotting shows that recombinant Tuf_Ab_ and Tuf_Lp_ bound plasminogen. Tuf_Sp_ served as a positive control, BSA as a negative control for unspecific binding.

Elongation factor Tuf is a conserved protein and various human pathogenic microbes utilize Tuf as a surface exposed plasminogen-binding protein [28–30]. Therefore, we speculated that Tuf of *A*. *baumannii* may also serve as a plasminogen-binding protein. Following PCR amplification, the respective fragment encoding the entire Tuf protein lacking the initial methionine residue was ligated into the pQE-30 Xa vector for the production of an N-terminally hexahistidine-tagged protein. For control purposes, the elongation factor Tuf of *Legionella pneumophila* (Tuf_Lp_) was amplified and cloned accordingly. Next, the *E*. *coli* produced and affinity purified proteins were subjected to SDS-PAGE. Silver staining and Westernblot analyses were performed to assess purity of the recombinant proteins. Tuf is a highly conserved protein, and an antiserum raised against Tuf of *S*. *pneumoniae* (anti-Tuf_Sp_), detects Tuf from various bacterial species. Using the Tuf_Sp_ antiserum, we were able to detect Tuf proteins of both *A*. *baumannii* (Tuf_Ab_) and *L*. *pneumophila* (Tuf_Lp_) (Fig 2A). Next, binding of plasminogen to the recombinant Tuf proteins was assessed using Far Western blotting. Tuf_Ab_, as well as Tuf_Lp_ and the positive control Tuf_Sp_ all bound human plasminogen (Fig 2B).

**Fig 3:**
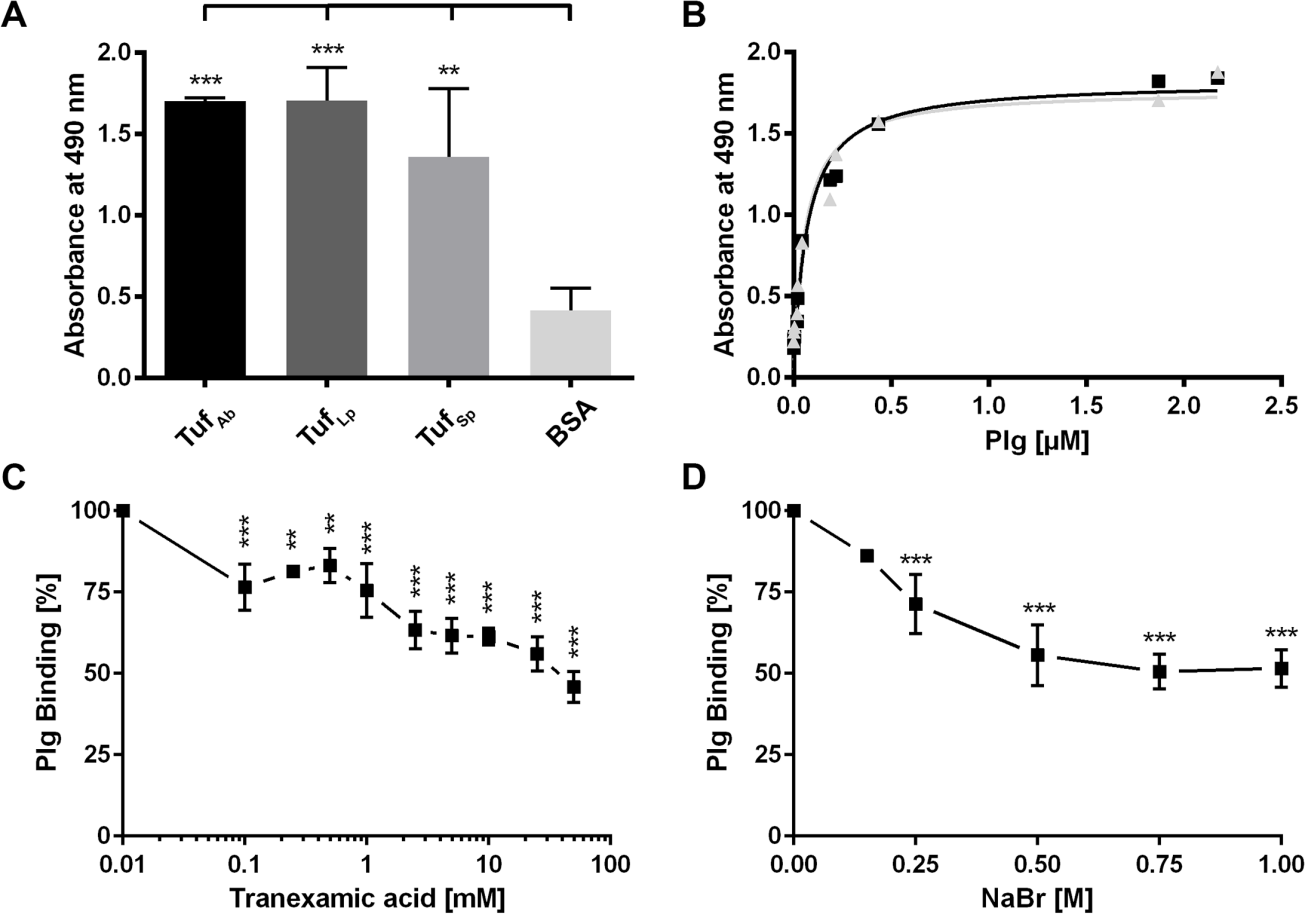
Further characterization of Tuf-Plasminogen interaction. (A) Binding of plasminogen (10 μg/ml) to immobilized recombinant Tuf proteins (5 μg/ml) derived from various species was assessed by ELISA. Tuf_Sp_ was used as a positive control, BSA as a negative control for nonspecific binding. Bound plasminogen was detected using a polyclonal plasminogen antiserum. (B) Binding of plasminogen to immobilized Tuf_Ab_ and Tuf_Lp_ occurred in a dose-dependent manner. Tuf proteins (5 μg/ml) were immobilized and incubated with increasing amounts of plasminogen. Binding of plasminogen was analyzed by ELISA using a polyclonal plasminogen antiserum. (C) Role of lysine residues in the Tuf_Ab_-plasminogen interaction. Binding of plasminogen (10 μg/ml) to immobilized Tuf_Ab_ was assayed by ELISA, using a polyclonal plasminogen antiserum, in the presence of increasing concentrations of the lysine analog tranexamic acid. (D) Impact of ionic strength on plasminogen binding to Tuf_Ab_. Tuf_Ab_ was immobilized and incubated with plasminogen (10 μg/ml) and increasing concentrations of NaBr. Plasminogen binding was analyzed by ELISA using a polyclonal plasminogen antiserum. Data represent means and standard deviation of at least three different experiments, each conducted in triplicate. **, p ≤ 0.01 and ***, p ≤ 0.001, one-way ANOVA with Bonferroni post hoc test.

We next sought to gain insight into the molecular protein-protein interaction. To determine whether Tuf_Ab_ was able to bind plasminogen under non-denaturing conditions, microtiter plates were coated with recombinant Tuf proteins or BSA as a control for unspecific binding (5 μg/ml) and binding of plasminogen was assayed by ELISA. In addition to Tuf_Sp_ which served as a positive control, both Tuf_Ab_ and Tuf_Lp_ bound plasminogen (Fig 3A) and binding to Tuf_Ab_ and Tuf_Lp_ occurred in a dose-dependent manner (Fig 3B). Using non-linear regression, the apparent dissociation constants (K_d_) for the Tuf-plasminogen interaction were determined to be in the low nanomolar range with K_d_ = 57 (± 15) nM for Tuf_Ab_ and K_d_ = 69 (± 15) nM for Tuf_Lp_.

### Influence of lysine residues and ionic strength on Tuf_Ab_-plasminogen interaction

Plasminogen interacts with a number of human receptors and bacterial proteins through lysine binding sites located within the kringle domains [36, 37]. To investigate the role of lysine residues in the Tuf_Ab_-plasminogen interaction, binding studies were conducted, using the lysine analog tranexamic acid. Addition of tranexamic acid significantly reduced the interaction between Tuf_Ab_ and plasminogen. 50 mM of tranexamic acid resulted in a 50% decrease of plasminogen binding to Tuf_Ab_ when compared to reactions without the lysine analog (Fig 3C). The positively charged ε-amino group of lysine residues suggests, that the Tuf_Ab_-plasminogen interaction could be susceptible to changes in ionic strength. To assess the role of ionic strength on binding of plasminogen, binding studies were performed in the presence of increasing concentrations of NaBr. The latter was used instead of NaCl to increase ionic strength, as the chloride anion promotes a closed conformation of plasminogen, which might adversely affect plasminogen binding irrespective of ionic strength. An increase in ionic strength through addition of NaBr had a significant effect on the Tuf_Ab_-plasminogen interaction. In the presence of 1 M NaBr, binding of plasminogen to Tuf_Ab_ was reduced to approximately 55% when compared to reactions where NaBr was omitted (Fig 3D).

### Conversion of Tuf-bound plasminogen to active plasmin

**Fig 4:**
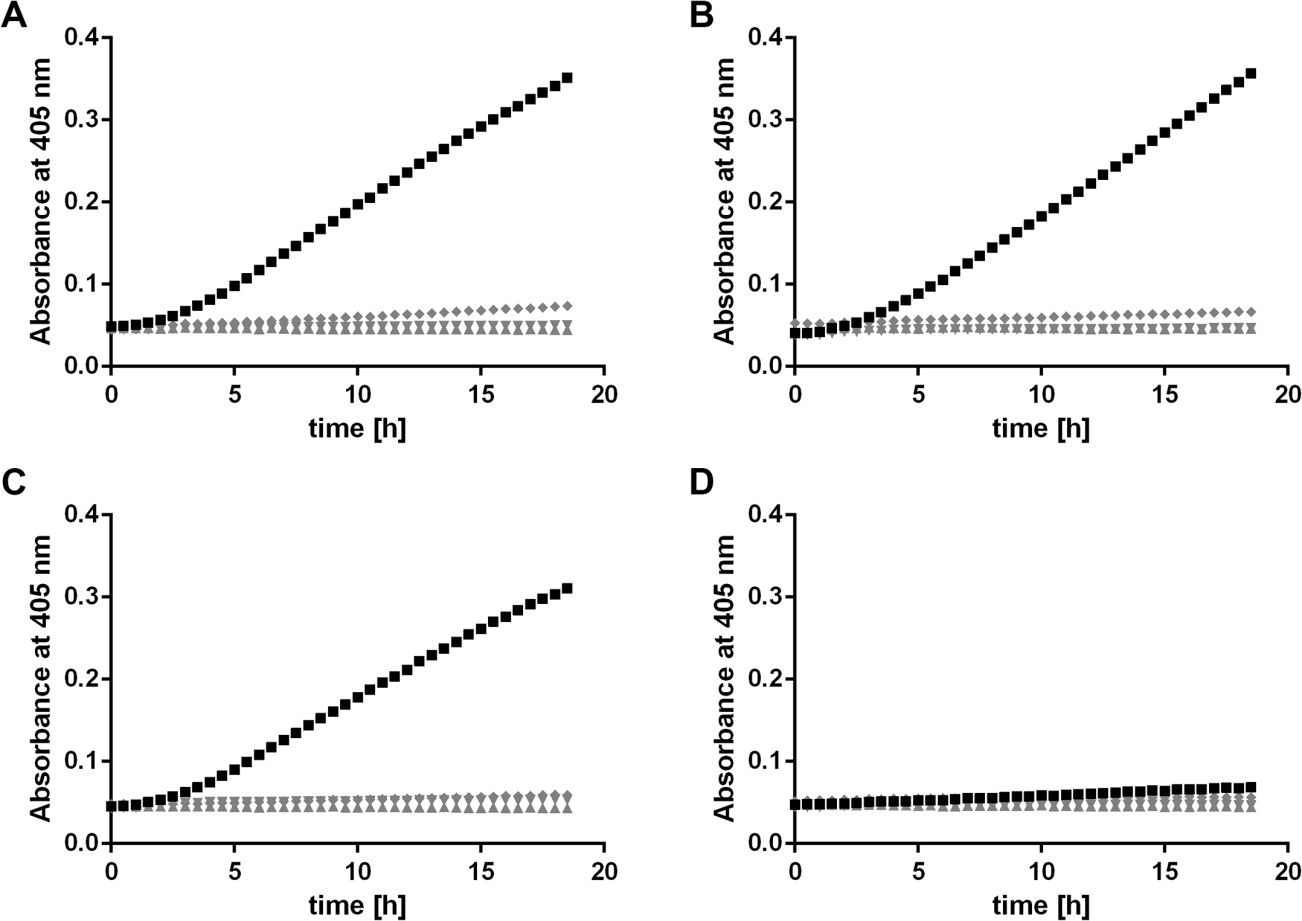
*A*. *baumannii* Tuf-bound plasminogen is converted to active plasmin by uPA. Microtiter plates were coated with 5 μg/ml of recombinant Tuf_Ab_ (A), Tuf_Lp_ (B), Tuf_Sp_ (C) or BSA as a negative control for unspecific binding (D) and incubated with plasminogen (10 μg/ml). Following several wash steps, a reaction mixture containing the plasminogen activator uPA (final concentration of 0.1 μg/ml) and the chromogenic substrate D-Val-Leu-Lys-*p*-nitroanilide dihydrochloride (S-2251) was added (■). Control reactions included 50 mM of the lysine analog tranexamic acid (♦) or omitted plasminogen (▼) or uPA (▲), respectively. Microtiter plates were incubated at RT for 18 h and absorbance at 405 nm was measured at 30 min intervals. At least three independent experiments were conducted, each in triplicate. Data shown are from a representative experiment. For clarity, graphs of negative controls are shaded gray.

Endogenous plasminogen activators such as tissue-type (tPA) or urokinase-type plasminogen activators (uPA) [15, 38], as well as bacterial molecules such as staphylokinase [39] or streptokinase [40, 41] convert plasminogen to the active serine protease plasmin. To determine whether Tuf-bound plasminogen is accessible to the plasminogen activator uPA, microtiter plates were coated with the respective Tuf proteins and after blocking, incubated with plasminogen. Following several wash steps, the plasminogen activator uPA was added together with the plasmin-specific chromogenic substrate D-Val-Leu-Lys-*p*-nitroanilide dihydrochloride (S-2251). In addition to the positive control Tuf_Sp_, plasminogen bound to Tuf_Ab_ and Tuf_Lp_ were accessible to uPA and subsequently converted to active plasmin (Fig 4). Additional control reactions either including the lysine analog tranexamic acid or omitting plasminogen or the plasminogen activator uPA, respectively, did not result in significant degradation of the chromogenic substrate.

### Degradation of fibrinogen by Tuf-bound plasmin

**Fig 5:**
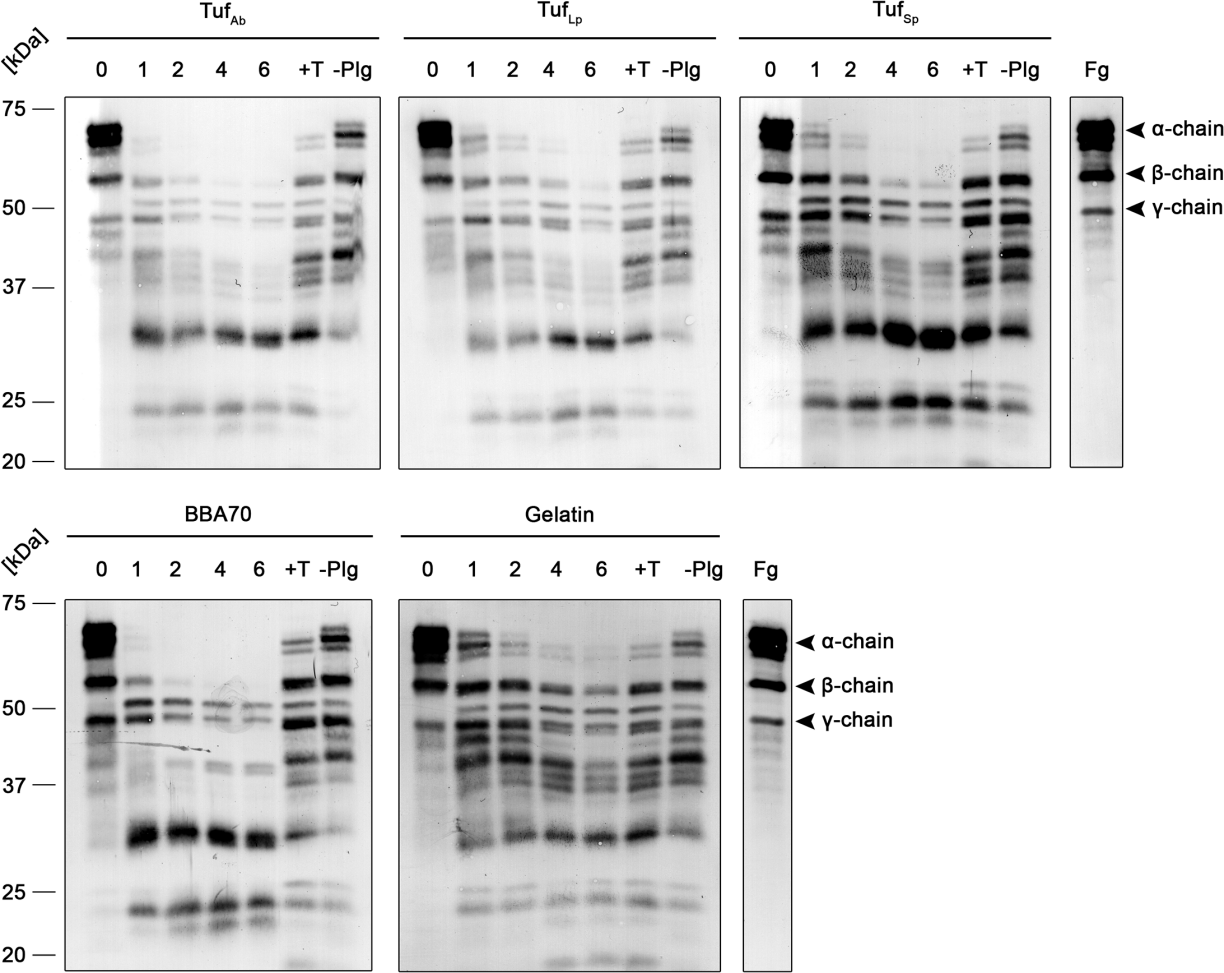
Degradation of fibrinogen by Tuf-bound plasmin. Tuf proteins, BBA70 and gelatin (5 μg/ml) were immobilized on microtiter plates, blocked and incubated with plasminogen (10 μg/ml). Following several wash steps, a reaction mixture containing the plasminogen activator uPA (0.16 μg/ml) and fibrinogen (20 μg/ml) was added and plates were incubated at 37°C. Samples were taken at the indicated time intervals and separated via SDS-PAGE. Upon transfer to nitrocellulose membranes, fibrinogen or its degradation products were detected in a Western blot analysis using a polyclonal fibrinogen antiserum. Controls included the lysine analog tranexamic acid (+T) and omission of plasminogen (-Plg). Fg, fibrinogen. Shown are representative results from several independent experiments.

Plasmin is the central component of the human fibrinolytic system and proteolytically degrades fibrin(ogen) [42]. Since Tuf-bound plasminogen is accessible to uPA and readily converted to active plasmin, we next sought to investigate, whether Tuf-bound plasmin retained its physiological activity and was able to cleave fibrinogen as its physiological substrate. Microtiter plates were coated with recombinant Tuf proteins or gelatin, blocked and incubated with plasminogen. After incubation, the activator uPA was added together with fibrinogen. Reactions were then incubated at 37°C and samples were taken at several time intervals. Following separation by SDS-PAGE, fibrinogen and its degradation products were detected with a polyclonal fibrinogen antiserum employing Western blot analysis. As shown in Fig 5, the fibrinogen α-chain was completely degraded following incubation for 1–4 h in reactions with Tuf_Ab_, Tuf_Lp_ as well as Tuf_Sp_. The plasminogen-binding protein BBA70 of *B*. *burgdorferi* served as an additional control, and for reactions with BBA70, degradation of fibrinogen was slightly more efficient, the fibrinogen α-chain was degraded after 1 h. For the Tuf proteins and BBA70, prominent degradation of the β-chain was also observed. Additionally, specific degradation products appeared over time. However, degradation was also observed with gelatin, which served as a negative control for unspecific binding. In addition, some degradation was also observed for control reactions including the lysine analog tranexamic acid and in control reactions omitting plasminogen altogether. Further control experiments were conducted to assess the stability of fibrinogen during prolonged incubation at 37°C and to analyze plasmin-mediated degradation in the absence of recombinant proteins (see S1 Fig) Fibrinogen remained stable when incubated for 24 h at 37°C. When incubated with plasminogen and uPA, fibrinogen was completely degraded after 2 h. Of note, degradation was also observed when fibrinogen was incubated with uPA in the absence of plasminogen.

### Tuf-bound plasmin degrades the complement opsonin C3b

**Fig 6:**
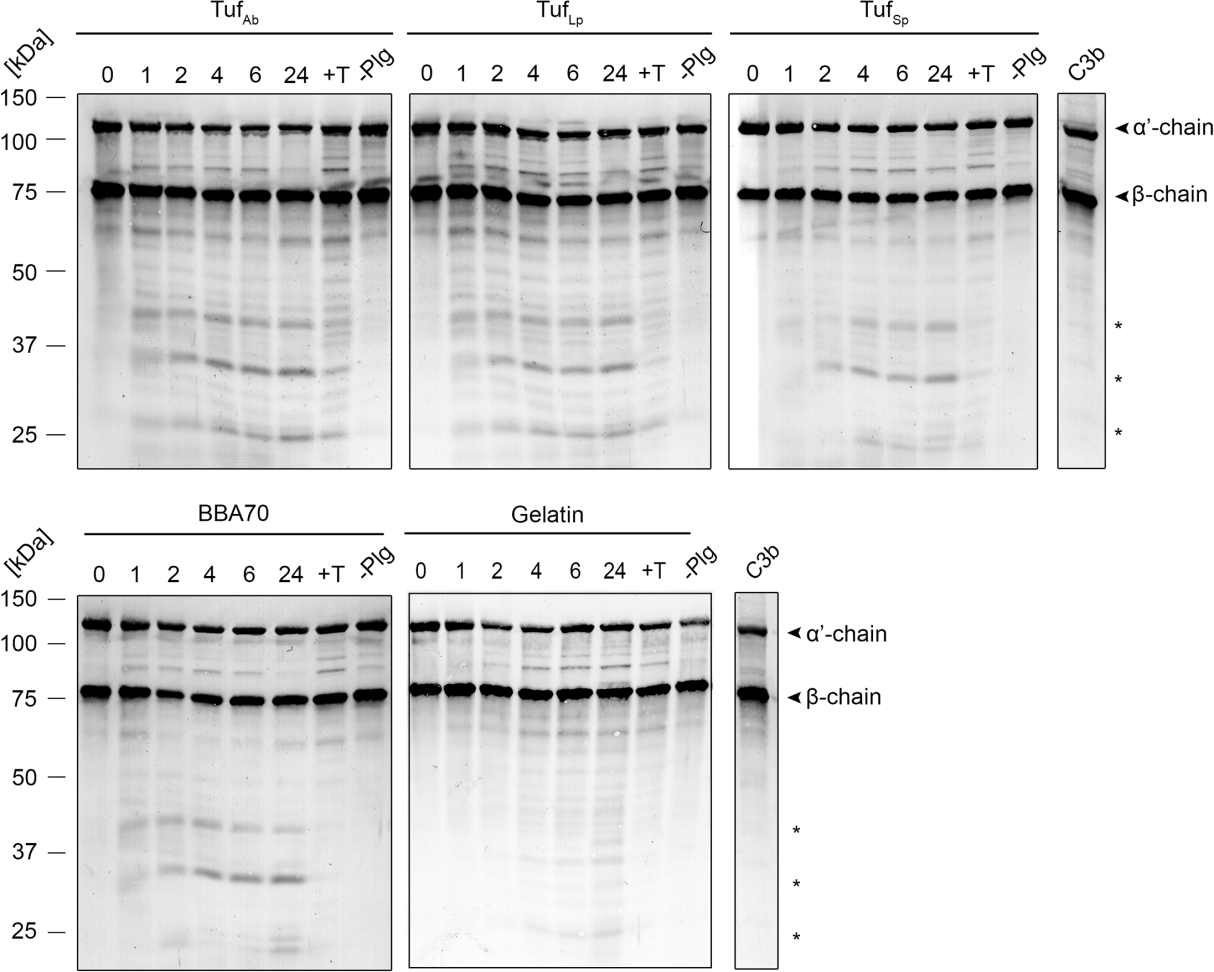
Tuf-bound plasmin degrades the complement opsonin C3b. Microtiter plates were coated with recombinant Tuf proteins (10 μg/ml), BBA70 or gelatin as a negative control for unspecific binding. Following incubation with plasminogen (20 μg/ml) and several wash steps, a reaction mixture containing the plasminogen activator uPA (0.16 μg/ml) and C3b (20 μg/ml) was added and microtiter plates were incubated at 37°C. Control reactions included the lysine analog tranexamic acid (+T) or omitted the incubation step with plasminogen (-Plg). Samples were taken at the indicated time intervals and separated by SDS-PAGE. C3b and its degradation products were detected by Western blot analysis probing the membranes with a polyclonal C3 antiserum. Degradation products with apparent molecular masses of approximately 43 kDa, 37 kDa, and 27 kDa are marked by asterisks. Results shown are representative of several independent experiments.

The serine protease plasmin exhibits a relatively broad substrate specificity and is able to degrade the key complement component C3b [16]. We thus decided to investigate whether Tuf-bound plasminogen is able to degrade C3b as well. Tuf proteins were immobilized onto microtiter plates and, after blocking, incubated with plasminogen. Wells were washed thoroughly and a reaction mixture containing both uPA and C3b was added. Plates were incubated at 37°C and samples were taken at different time intervals. Following separation of proteins by SDS-PAGE and transfer to nitrocellulose membranes, a polyclonal antiserum raised against C3 was used to detect C3b and its degradation products. As shown in Fig 6, plasmin, bound to Tuf_Ab_, Tuf_Lp_ and the positive controls Tuf_Sp_ and BBA70, was able to degrade C3b as seen by the appearance of specific degradation products with molecular masses of approximately 43 kDa, 37 kDa and 27 kDa over time. Some degradation of C3b was also observed for reactions with gelatin. Interestingly, in case of the Tuf proteins, degradation products could be observed in control reactions with tranexamic acid, while no degradation was observed when plasminogen was omitted. To assay the stability of C3b over the prolonged incubation period at 37°C and to assess degradation of C3b by plasmin in the absence of recombinant proteins, further experiments were performed. S2 Fig shows that C3b remained stable over 24 h at 37°C. C3b was degraded by plasmin(ogen) in the presence of uPA. In the absence of uPA, no degradation of C3b was observed. Incubation of C3b with factor I in the presence of factor H (C3b +FH +FI) resulted in the generation of degradation products with apparent molecular masses of 68 kDa, 43 kDa, and 27 kDa, which are distinct from the C3b cleavage fragments generated by plasmin.

### Tuf_Ab_ is exposed on the surface of *A*. *baumannii*

**Fig 7:**
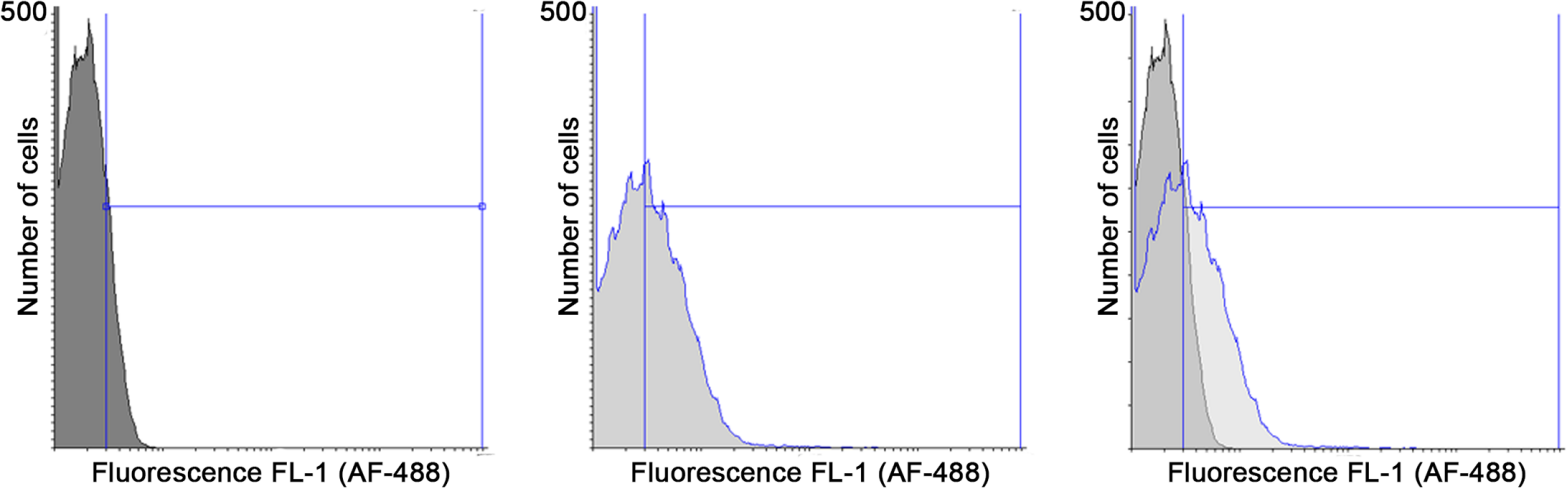
Localization of Tuf_Ab_ on the outer surface of *A*. *baumannii* ATCC 19606. Late log-phase *A*. *baumannii* cells (5 x 10^8^) were harvested and resuspended in PBS with 1% (w/v) BSA to block unspecific binding sites. Cells were then incubated with a cross-reacting, polyclonal Tuf_Sp_ antiserum (1:10). Following several wash steps, cells were incubated with an Alexa Fluor 488 anti-rabbit conjugate (1:25). After incubation, cells were washed again and fixated with 3.75% PFA. Surface exposure of Tuf_Ab_ was then assayed using flow cytometry. 50,000 events were counted and approximately 40% (± 4.9%) of *A*. *baumannii* cells stained positive for Tuf_Ab_. Shown are representative results of three separate experiments.

Regarding surface exposure, it has been previously shown, using immune electron microscopy and Western blotting, that Tuf_Ab_ can be associated both with the bacterial surface and outer membrane vesicles of *A*. *baumannii* [33]. We sought to confirm these findings, using the cross-reacting Tuf_Sp_ antiserum in flow cytometry experiments. Late log-phase *A*. *baumannii* cells were washed thoroughly, blocked and incubated with the Tuf_Sp_ antiserum. Following more wash steps, cells were incubated with an Alexa Fluor 488-conjugated anti-rabbit antibody. After incubation, cells were fixated with PFA and analyzed by flow cytometry. Approximately 40% (± 4.9%) of *A*. *baumannii* cells stained positive for Tuf_Ab_ (Fig 7).

### Tuf proteins are highly conserved

**Fig 8:**
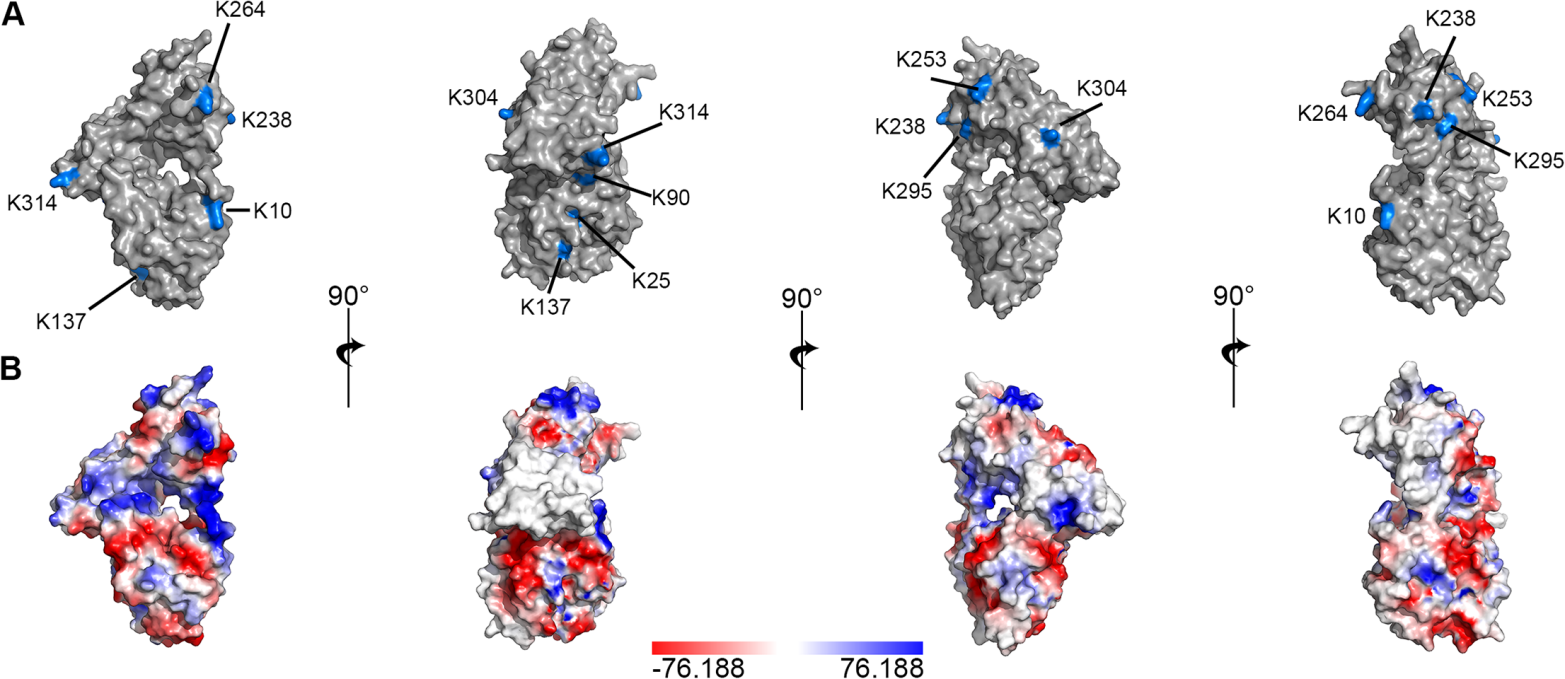
Conserved lysine residues of elongation factor Tuf and charge distribution. 3D-structure of Tuf of *E*. *coli* in its GDP-bound state. (A) Lysine residues conserved among the organisms analyzed in S3 Fig are highlighted in blue. Note that residues K3 and K5 from sequence alignment are missing, as the PDB file did not include those amino acids. (B) Predicted charge distribution across the Tuf protein. Fig was created using PyMOL, Version 1.3 and is based on PDB file 2FX3 [45].

Elongation factor Tuf is a highly conserved protein [43, 44] and sequence analysis of the Tuf proteins of *A*. *baumannii*, *L*. *pneumophila*, *S*. *pneumoniae*, *P*. *aeruginosa*, *L*. *interrogans*, and *E*. *coli* (see S3 Fig) revealed amino acid sequence identities ranging from 67% and 85%. Overall, twelve conserved lysine residues were identified. Surface exposed lysine residues might potentially interact with plasminogen. Fig 8A shows the 3D-structure of elongation factor Tuf of *E*. *coli* [45]. Conserved lysine residues are highlighted in blue. The predicted charge distribution of the *E*. *coli* Tuf protein is shown in Fig 8B. While not all lysine residues are located in areas of the protein with a net positive charge, the lysine residues K10, K264, and K314 fall within positively charged regions of the *E*. *coli* Tuf protein.

## Discussion

*A*. *baumannii* has been emerging as a successful pathogen [46] and many factors contribute to the survival of *A*. *baumannii* in the hospital environment, including its remarkable resistance to desiccation [5] and to major antimicrobial drugs [6]. Owing to the fact that *A*. *baumannii* is an opportunistic pathogen that mainly affects immunocompromised patients, attributable mortality rates are difficult to assess and range from 8–35% [47]. Beyond tolerance to desiccation and resistance to antibiotics, a number of mechanisms seem to contribute to the pathogenic potential of *A*. *baumannii* [48] but not all of them are well understood. Interestingly, many of the virulence factors described to date are involved in the serum resistance of *A*. *baumannii*.

It has been previously shown that certain *A*. *baumannii* isolates are resistant to complement-mediated killing, however the mechanisms involved in serum resistance are still poorly understood. Many serum-resistant strains of *A*. *baumannii* form biofilms, however formation of biofilms does not seem to be a prerequisite for survival in human serum [49]. One strategy used by many pathogens to overcome the bactericidal effects of human serum is the acquisition of fluid phase complement regulators from the host, for example factor H, the key complement regulator of the alternative pathway. While it has been clearly shown that *A*. *baumannii* activates primarily the alternative pathway [49, 50], the data regarding interaction with factor H are conflicting. While one study identified the outer membrane protein OmpA as a factor H binding protein [50], a second study demonstrated that viable *A*. *baumannii* cells do not in fact bind this complement regulator [49]. The latter results are in line with our own findings indicating that clinical isolates of *A*. *baumannii* did not bind factor H (data not shown) and, thus would indicate that *A*. *baumannii* employs other strategies to survive in human serum.

Here we showed for the first time, that *A*. *baumannii* binds human plasminogen and we identified Tuf_Ab_ as a plasminogen-binding protein. While plasmin(ogen) is a key component of the fibrinolytic system, it can also function as a complement regulator [16]. Recombinant Tuf_Ab_ binds human plasminogen both under denaturing and non-denaturing conditions (see Figs 2 and 3) and the intensity of binding is comparable to Tuf proteins derived from other pathogens (Tuf_Sp_ and Tuf_Lp_). Tuf_Ab_ binds plasminogen dose-dependently and with an apparent dissociation constant of K_d_ = 57 ± 15 nM. Thus *A*. *baumannii* Tuf binds plasmionogen with a rate constant comparable to that of other bacterial plasminogen-binding proteins, such as enolase or DnaK of *Bifidobacterium animalis* with dissociation constants of 42 nM or 11 nM, respectively [51]. Several pathogenic bacteria bind plasminogen and similarly as described here for *A*. *baumannii*, other bacterial Tuf proteins were previously characterized as plasminogen binding proteins, e.g. Tuf of *M*. *tuberculosis* [31], *P*. *aeruginosa* [28], *L*. *interrogans* [30] and *S*. *pneumoniae* [29], thus demonstrating the significance of Tuf as a potential virulence factor.

Binding of plasminogen to Tuf_Ab_ is at least partially dependent on lysine residues and ionic strength (see Fig 3). The lysine analog tranexamic acid significantly reduced plasminogen binding to Tuf_Ab_ however even at 50 mM tranexamic acid, binding levels remained at approximately 50%. This suggests, that other factors influence plasminogen binding to Tuf_Ab_ and this result is in contrast to other bacterial plasminogen-binding proteins such as Lpd of *P*. *aeruginosa* [52] or PE of *H*. *influenzae* [20]. Of note, human factor Xa interacts with the N-terminus of plasminogen independently of lysine residues [53], however factor Xa also interacts in a lysine dependent fashion with the C-terminal kringle domains of plasminogen. At neutral pH, lysine residues carry a net positive charge, thus an increase in ionic strength should affect plasminogen binding. Indeed, an increase in the ionic strength to 1 M NaBr, reduced plasminogen binding by approximately 45%. NaBr was used in these experiments, because chloride anions promote the closed conformation of the plasminogen molecule [54] and, thus could negatively influence the Tuf_Ab_-plasminogen interaction. When comparing the Tuf proteins of various microorganisms, we identified twelve conserved lysine residues (see S3 Fig), at least three of which are located in areas of the Tuf protein with a net positive charge (Fig 8), making it tempting to speculate that those residues might be involved in ionic interactions with plasminogen. The findings regarding the influence of ionic strength would support the notion that binding of plasminogen by Tuf_Ab_ is only partially dependent on ionic interactions mediated by lysine residues, and that other, non-ionic interactions may contribute to plasminogen binding. Findings published for Tuf_Sp_ are in line with our own data for Tuf_Ab_, showing that plasminogen binding is at least partially dependent on ionic strength [29]. On the other hand, Tuf of *L*. *interrogans* binds plasminogen independently of ionic interactions [30].

Plasminogen bound to Tuf proteins is accessible to the plasminogen activator uPA and proteolytically active plasmin was generated, as demonstrated by cleavage of the chromogenic substrate D-Val-Leu- Lys-*p*-nitroanilide dihydrochloride (Fig 4). More importantly, Tuf-bound plasmin was able to degrade the physiological substrate fibrinogen (Fig 5). Degradation of fibrinogen was also observed in control reactions with the lysine analog tranexamic acid. This is in line with the binding studies showing that tranexamic acid did not completely inhibit binding of plasminogen to Tuf. Clearly, the reduced amount of plasminogen that is still bound to Tuf even in the presence of tranexamic acid, once converted to plasmin, is still sufficient to degrade fibrinogen. Of note, degradation was also observed in reactions with gelatin, used as a control for unspecific binding, though degradation seemed to be limited to the fibrinogen α-chain, while the β- and γ-chain remained intact. Additionally, some degradation of the fibrinogen α-chain was observed in reactions omitting plasminogen altogether. To determine whether this degradation is due to unspecific binding, the long incubation period (6 h) or contamination of uPA with plasminogen, additional control experiments were performed (see S1 Fig). These experiments revealed that fibrinogen remained stable when incubated at 37°C for 24 h. However, when fibrinogen was incubated with uPA in the absence of plasminogen, some degradation occurred, suggesting that trace amounts of plasminogen were present in the uPA preparation used in these experiments. The resulting level of “background” degradation means that results of the fibrinogen degradation assay must be interpreted very carefully, though the fact that degradation was generally stronger in reactions with Tuf proteins and with BBA70-bound plasmin still suggests that plasmin bound to these proteins retains its proteolytic activity.

It has been proposed that fibrin can trap invading pathogenic microorganisms at the site of entry, thus preventing their spread through the bloodstream [55]. For *S*. *canis*, it has been shown that binding of plasminogen to the SCM protein and subsequent conversion to plasmin promotes both degradation of fibrinogen as well as transmigration through thrombi [56]. *A*. *baumannii* secretes the CpaA protease which is able to cleave fibrinogen and deregulates blood coagulation [57]. It is tempting to speculate, that binding of plasminogen by Tuf_Ab_ and subsequent conversion to plasmin might provide *A*. *baumannii* with a second proteolytic activity and potentially aid the bacteria in dissemination.

In addition to degradation of fibrinogen, plasmin is also able to cleave several complement components, including C3b and C5 [16]. Tuf- and BBA70-bound plasminogen, upon conversion to plasmin by uPA, degraded C3b, as shown by the appearance of specific degradation products over time (Fig 6). Again, for reactions with Tuf proteins, C3b degradation was also observed in the presence of tranexamic acid, thus demonstrating that the reduced levels of bound plasminogen were still able to cleave this complement component upon activation to plasmin. Some degradation of C3b was also seen for the negative control gelatin, but this was significantly less prominent when compared to reactions with BBA70 or the Tuf proteins. When plasminogen was omitted, no degradation of C3b was observed. This is in contrast to the fibrinogen degradation assay, even though the uPA preparation used was the same in both assays. Conceivably, this is due to the fact that fibrinogen, as the physiological substrate, is considerably more susceptible to degradation by plasmin, hence trace amounts of plasmin would degrade fibrinogen much more efficiently than C3b, even though incubation times were longer in the C3b degradation assay. Analogous to the fibrinogen degradation assay, additional control experiments were performed (see S2 Fig). Data from these experiments confirmed that C3b remained stable when incubated at 37°C for 24 h and that no degradation of C3b occurred when incubated with uPA in the absence of plasminogen.

Several other pathogenic microorganisms acquire plasminogen and upon conversion to plasmin use the proteolytic activity to cleave complement components, for example *H*. *influenzae* [20] and *L*. *interrogans* [58]. *A*. *baumannii* efficiently activates the alternative pathway of complement [49, 50] and while a serum-sensitive isolate showed deposition of C3, deposition was reduced on a serum-resistant isolate [49]. The serine protease PKF is secreted by *A*. *baumannii* and is able to specifically inhibit the alternative pathway of complement [59], though the mode of action remains unclear. Acquisition of plasminogen and subsequent conversion to plasmin could provide *A*. *baumannii* with further proteolytic activity, allowing for cleavage of the key complement component C3b, possibly supplementing endogenous proteases and thereby enhancing resistance to complement-mediated killing.

Tuf_Ab_ is associated with the cell surface and with outer membrane vesicles of *A*. *baumannii* [33]. Using flow cytometry, surface exposure of Tuf_Ab_ was confirmed (Fig 7). Surface exposure of Tuf_Ab_ is a prerequisite for interaction with host proteins. In addition to binding plasminogen, Tuf_Ab_ also interacts with fibronectin [33] and peptides derived from Tuf_Ab_ as well as Tuf of *E*. *coli* have been shown to interact with the periplasmic *A*. *baumannii* dithiol oxidase DsbA [60]. Considering the cytoplasmic functions of Tuf proteins in general [26, 27], the elongation factor Tuf clearly is a multifunctional bacterial moonlighting protein.

Ventilator-associated pneumonia is one of the infections caused by *A*. *baumannii* that is associated with the highest mortality rates [4]. Another pathogen causing lung infection is *L*. *pneumophila*, the causative agent of Legionnaires’ disease [61]. In the present study we also investigated Tuf of *L*. *pneumophila*. Similar to TufAb, TufLp was able to bind plasminogen and upon conversion to plasmin, degrade both fibrinoigen and C3b, providing further evidence that moonlighting plasminogen-binding proteins are employed by a large number of pathogenic microorganisms. *L*. *pneumophila* also expresses an outer membrane protein homologous to Pla of *Yersinia pestis*, which is able to convert plasminogen to proteolytically active plasmin [62], and may be involved in penetration of alveolar epithelial barriers and basement membranes. Furthermore, while it has been clearly established that *L*. *pneumophila* replicates in free living amoeba [63], it has more recently been demonstrated that *A*. *baumannii* can also be isolated from amoeba [64], and that amoeba protect intracellular bacteria from adverse conditions, facilitating survival in hospital water networks [65, 66].

The concept of moonlighting proteins, where a single protein is performing more than one function, is becoming increasingly more accepted [67] and is starting to replace the paradigm, that one gene equals one protein, equals a single function. Tuf is exposed on the surface of a number of bacterial pathogens, where it interacts with various human serum proteins. The Tuf proteins of *L*. *interrogans*, *P*. *aeruginosa* and *S*. *pneumoniae* are all surface exposed, moonlighting proteins, and function as bacterial ligands for the human serum proteins plasminogen and factor H, and in the case of the latter two also factor H like protein-1 (FHL-1) and factor H related protein-1 (FHR-1) [28–30].

In general, the synthetic cost of extracellular proteins in bacteria is reduced compared to cytoplasmic or inner membrane proteins [68], as they cannot be recycled by the bacterial cell. Similarly to enolase of *B*. *burgdorferi*, which is another example of a moonlighting plasminogen-binding protein [69], Tuf_Ab_ is rich in energetically less expensive amino acids, such as alanine (8.5%), glycine (9.4%) while energetically expensive amino acids such as tyrosine (3.5%), phenylalanine (2.9%) and tryptophan (0%) occur less frequently. The average synthetic cost for Tuf_Ab_ is 22.5 ATP/aa, which is somewhat higher than the average cost of extracellular proteins. However, the fact that Tuf seems to have multiple functions both in the cytoplasm and when associated with the cell surface could make it more economical for the bacterial cell than having to synthesize a single protein for each individual function.

In conclusion, we show for the first time that *A*. *baumannii* binds human plasminogen and identify Tuf_Ab_ as a cell surface localized plasminogen-binding protein of *A*. *baumannii*. Plasminogen bound to Tuf_Ab_ can be converted to proteolytically active plasmin which degrades both fibrinogen and the key complement component C3b. Tuf_Ab_ may thus play a role in virulence and contribute to both dissemination and serum resistance of *A*. *baumannii*.

## Supporting Information

S1 Fig Stability of fibrinogen and degradation by plasmin. To assess whether degradation of fibrinogen occurs during prolonged incubation at 37°C, purified fibrinogen was incubated for 24 h (Fg (24 h)). Furthermore, fibrinogen (20 μg/ml) was incubated with the activator uPA (0.16 μg/ml) either in the absence (Fg–Plg +uPA) or in the presence of 10 μg/ml plasminogen (Fg +Plg +uPA), in a total volume of 100 μl 50 mM Tris/HCl pH 7.5. Reactions were incubated for 2 h at 37°C. Following incubation, samples were separated via SDS-PAGE and blotted onto nitrocellulose. The membrane was probed with an antiserum raised against fibriniogen (1:1000) to visualize fibrinoigen or its degradation products. Purified fibrinogen (500 ng) served as an additional control.
(TIF)

S2 Fig Stability of C3b and degradation by plasmin and factor H. To determine the stability of C3b over prolonged incubation at 37°C, purified C3b was incubated for 24 h (C3b (24h)). Degradation of C3b by factor I in the presence of factor H was also assessed. C3b (20 μg/ml) was incubated with factor H (10 μg/ml, FH) and factor I (5 μg/ml, FI) in a total volume of 100 μl 50 mM Tris/HCl pH 7.5 for 2 h at 37°C. Additionally, C3b (20 μg/ml) was incubated with uPA (0.16 μg/ml) either in the absence (C3b –Plg +uPA) or in the presence of 10 μg/ml plasminogen (C3b +Plg +uPA) in a total volume of 100 μl 50 mM Tris/HCl pH 7.5 for 2 h at 37°C. Samples were separated by SDS-PAGE and transferred to a nitrocellulose membrane. C3b and its degradation products were detected by a polyclonal antiserum raised against C3. Purified C3b (500 ng) served as an additional control.
(TIF)

S3 Fig Amino acid sequence alignment of Tuf proteins. Amino acid sequences of Tuf proteins from *A*. *baumannii* (AIS05611.1), *L*. *pneumophila* (YP_094371.1), *S*. *pneumoniae* (ABJ53652.1), *P*. *aeruginosa* (AJD61976.1), *L*. *interrogans* (AAS71428.1) and *E*. *coli* (EDU63199.1), were aligned with Clustal Omega (1.2.1) and analysis with Clustal 2.1 revealed sequence identities ranging from 67% to 85%. Overall, twelve conserved lysine residues could be identified (shaded in black).
(TIF)
