# Correction: Translation Elongation Factor Tuf of *Acinetobacter baumannii* Is a Plasminogen-Binding Protein

**DOI:** 10.1371/journal.pone.0138398

**Published:** 2015-09-14

**Authors:** Arno Koenigs, Peter F. Zipfel, Peter Kraiczy

The images for S1 Fig, S2 Fig and S3 Fig are swapped. The image for S1 Fig should be S3 Fig, the image for S2 Fig should be S1 Fig, and the image for S3 Fig should be S2 Fig. Please view the correct Supporting Information Figures below.

S1 Fig Stability of fibrinogen and degradation by plasmin. To assess whether degradation of fibrinogen occurs during prolonged incubation at 37°C, purified fibrinogen was incubated for 24 h (Fg (24 h)). Furthermore, fibrinogen (20 μg/ml) was incubated with the activator uPA (0.16 μg/ml) either in the absence (Fg–Plg +uPA) or in the presence of 10 μg/ml plasminogen (Fg +Plg +uPA), in a total volume of 100 μl 50 mM Tris/HCl pH 7.5. Reactions were incubated for 2 h at 37°C. Following incubation, samples were separated via SDS-PAGE and blotted onto nitrocellulose. The membrane was probed with an antiserum raised against fibriniogen (1:1000) to visualize fibrinoigen or its degradation products. Purified fibrinogen (500 ng) served as an additional control.
(TIF)

S2 Fig Stability of C3b and degradation by plasmin and factor H. To determine the stability of C3b over prolonged incubation at 37°C, purified C3b was incubated for 24 h (C3b (24h)). Degradation of C3b by factor I in the presence of factor H was also assessed. C3b (20 μg/ml) was incubated with factor H (10 μg/ml, FH) and factor I (5 μg/ml, FI) in a total volume of 100 μl 50 mM Tris/HCl pH 7.5 for 2 h at 37°C. Additionally, C3b (20 μg/ml) was incubated with uPA (0.16 μg/ml) either in the absence (C3b –Plg +uPA) or in the presence of 10 μg/ml plasminogen (C3b +Plg +uPA) in a total volume of 100 μl 50 mM Tris/HCl pH 7.5 for 2 h at 37°C. Samples were separated by SDS-PAGE and transferred to a nitrocellulose membrane. C3b and its degradation products were detected by a polyclonal antiserum raised against C3. Purified C3b (500 ng) served as an additional control.
(TIF)

S3 Fig Amino acid sequence alignment of Tuf proteins. Amino acid sequences of Tuf proteins from A. baumannii (AIS05611.1), L. pneumophila (YP_094371.1), S. pneumoniae (ABJ53652.1), P. aeruginosa (AJD61976.1), L. interrogans (AAS71428.1) and E. coli (EDU63199.1), were aligned with Clustal Omega (1.2.1) and analysis with Clustal 2.1 revealed sequence identities ranging from 67% to 85%. Overall, twelve conserved lysine residues could be identified (shaded in black).
(TIF)
