# Supplementary figures and images for: Translation Elongation Factor Tuf of Acinetobacter baumannii Is a Plasminogen-Binding Protein
Source: PLoS One. 2015 Jul 31;10(7):e0134418. doi: 10.1371/journal.pone.0134418 (PMC4521846; doi:10.1371/journal.pone.0134418)

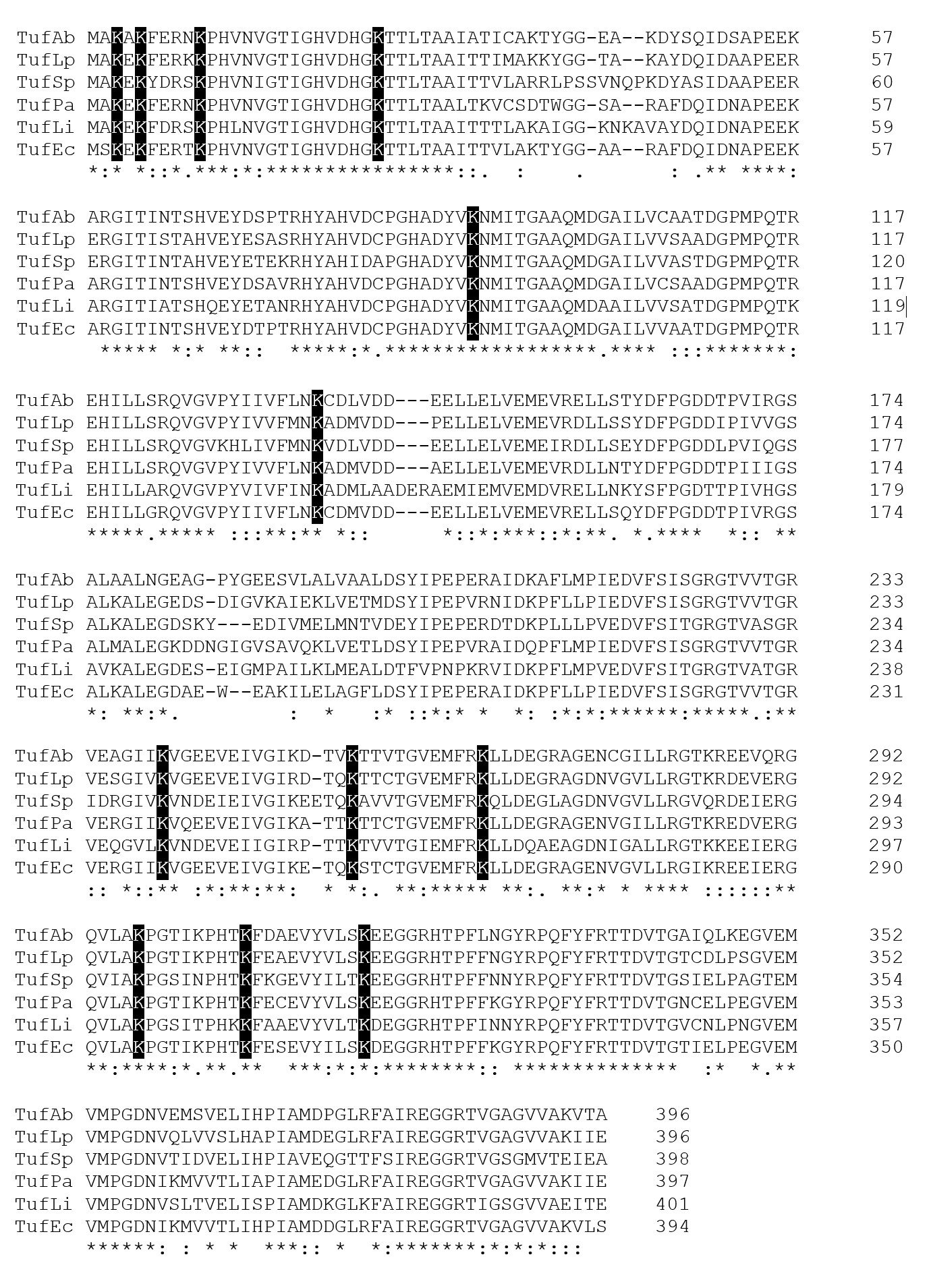

Supplement: S1 Fig — To assess whether degradation of fibrinogen occurs during prolonged incubation at 37°C, purified fibrinogen was incubated for 24 h (Fg (24 h)). Furthermore, fibrinogen (20 μg/ml) was incubated with the activator uPA (0.16 μg/ml) either in the absence (Fg–Plg +uPA) or in the presence of 10 μg/ml plasminogen (Fg +Plg +uPA), in a total volume of 100 μl 50 mM Tris/HCl pH 7.5. Reactions were incubated for 2 h at 37°C. Following incubation, samples were separated via SDS-PAGE and blotted onto nitrocellulose. The membrane was probed with an antiserum raised against fibriniogen (1:1000) to visualize fibrinoigen or its degradation products. Purified fibrinogen (500 ng) served as an additional control. (TIF) [file pone.0134418.s001.tif]

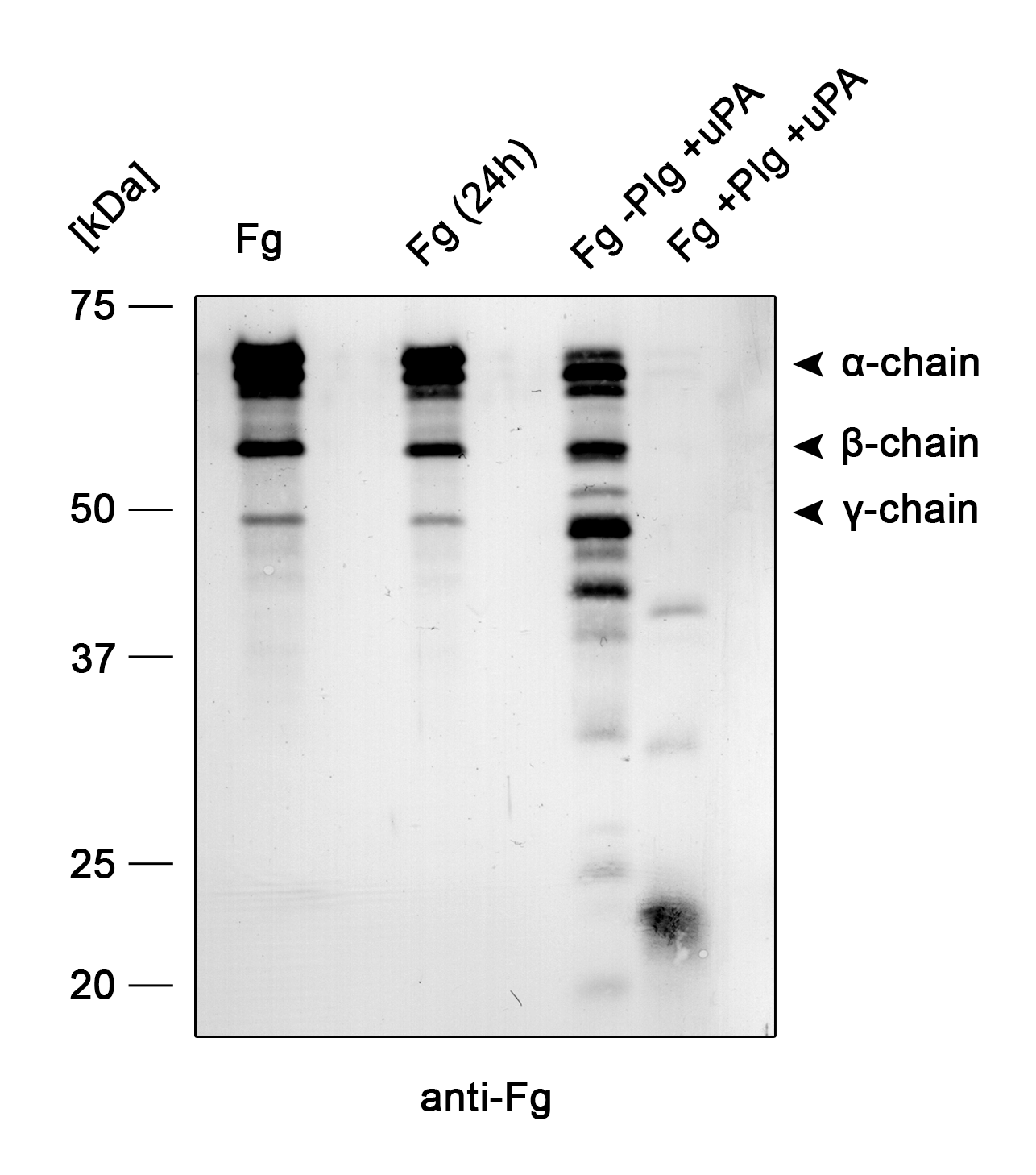

Supplement: S2 Fig — To determine the stability of C3b over prolonged incubation at 37°C, purified C3b was incubated for 24 h (C3b (24h)). Degradation of C3b by factor I in the presence of factor H was also assessed. C3b (20 μg/ml) was incubated with factor H (10 μg/ml, FH) and factor I (5 μg/ml, FI) in a total volume of 100 μl 50 mM Tris/HCl pH 7.5 for 2 h at 37°C. Additionally, C3b (20 μg/ml) was incubated with uPA (0.16 μg/ml) either in the absence (C3b –Plg +uPA) or in the presence of 10 μg/ml plasminogen (C3b +Plg +uPA) in a total volume of 100 μl 50 mM Tris/HCl pH 7.5 for 2 h at 37°C. Samples were separated by SDS-PAGE and transferred to a nitrocellulose membrane. C3b and its degradation products were detected by a polyclonal antiserum raised against C3. Purified C3b (500 ng) served as an additional control. (TIF) [file pone.0134418.s002.tif]

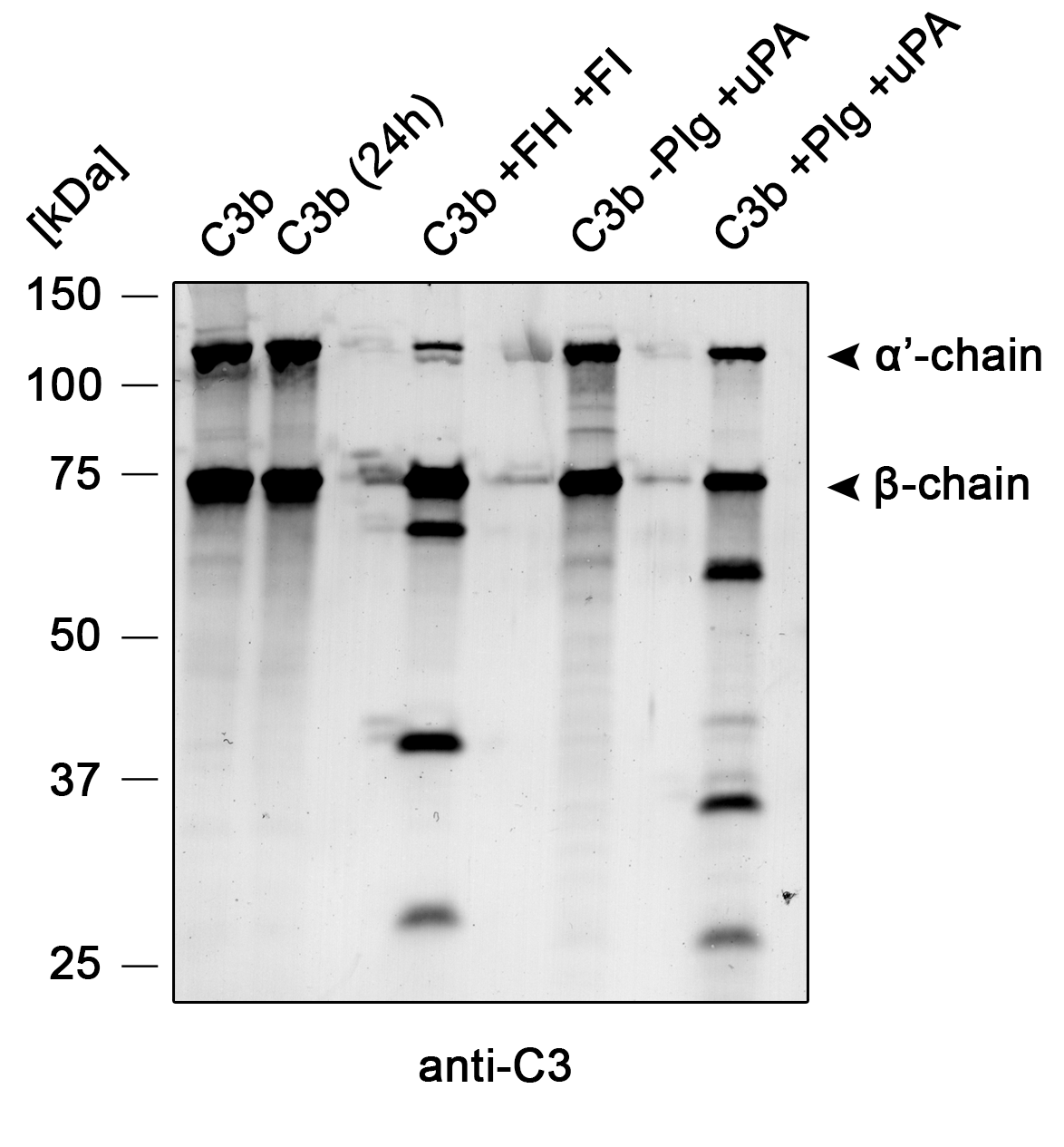

Supplement: S3 Fig — Amino acid sequences of Tuf proteins from A. baumannii (AIS05611.1), L. pneumophila (YP_094371.1), S. pneumoniae (ABJ53652.1), P. aeruginosa (AJD61976.1), L. interrogans (AAS71428.1) and E. coli (EDU63199.1), were aligned with Clustal Omega (1.2.1) and analysis with Clustal 2.1 revealed sequence identities ranging from 67% to 85%. Overall, twelve conserved lysine residues could be identified (shaded in black). (TIF) [file pone.0134418.s003.tif]
